# Thermogenesis, Flowering and the Association with Variation in Floral Odour Attractants in *Magnolia sprengeri* (Magnoliaceae)

**DOI:** 10.1371/journal.pone.0099356

**Published:** 2014-06-12

**Authors:** Ruohan Wang, Sai Xu, Xiangyu Liu, Yiyuan Zhang, Jianzhong Wang, Zhixiang Zhang

**Affiliations:** 1 National Engineering Laboratory for Tree Breeding, Key Laboratory for Genetics and Breeding of Forest Trees and Ornamental Plants, Ministry of Education, College of Biological Sciences and Biotechnology, Beijing Forestry University,Beijing, China; 2 Lab of Systematic Evolution and Biogeography of Woody Plants, College of Nature Conservation, Beijing Forestry University,Beijing, China; 3 School of Environment, Tsinghua University, Beijing, China; INRA-UPMC, France

## Abstract

*Magnolia sprengeri* Pamp. is an ornamentally and ecologically important tree that blooms at cold temperatures in early spring. In this study, thermogenesis and variation in the chemical compounds of floral odours and insect visitation in relation to flowering cycles were studied to increase our understanding of the role of floral thermogenesis in the pollination biology of *M. sprengeri*. There were five distinct floral stages across the floral cycle of this species: pre-pistillate, pistillate, pre-staminate, staminate and post-staminate. Floral thermogenesis during anthesis and consisted of two distinct peaks: one at the pistillate stage and the other at the staminate stage. Insects of five families visited *M. sprengeri* during the floral cycle, and sap beetles (*Epuraea* sp., Nitidulidae) were determined to be the most effective pollinators, whereas bees (*Apis cerana*, Apidae) were considered to be occasional pollinators. A strong fragrance was released during thermogenesis, consisting of 18 chemical compounds. Although the relative proportions of these compounds varied at different floral stages across anthesis, linalool, 1-iodo-2-methylundecane and 2,2,6-trimethyl-6-vinyltetrahydro-2H-pyran-3-ol were dominant. Importantly, we found that the floral blends released during the pistillate and staminate stages were very similar, and coincided with flower visitation by sap beetles and the two thermogenic episodes. Based on these results, we propose that odour acts as a signal for a reward (pollen) and that an odour mimicry of staminate-stage flowers occurs during the pistillate stage.

## Introduction

Recent studies have confirmed that thermogenic activity occurs in the reproductive organs of some flowering plants and is crucial for various phases of sexual reproduction, insect–plant interactions and the adaptation of plants to environmental conditions [1]. Thermogenesis is not a by-product of floral development but has been suspected to serve specific functions such as the volatilisation of floral fragrances [2],[3], enhanced blooming in cool weather [4], inflorescence unfolding, pollen maturation or release [5], provision of a warm environment for pollinating insects that temporarily reside in the flower [6],[7] and facilitation of fertilisation and seed set [8].

Thermogenic activity occurs in various structures of many plant families, including the cones of gymnosperms (Cycadaceae and Zamiaceae) and flowers or inflorescences of some angiosperms (Annonaceae, Araceae, Arecaceae, Aristolochiaceae, Cyclanthaceae, Hydnoraceae, Illiceaceae, Magnoliaceae, Nelumbonaceae, Nymphaeaceae, Rafflesiaceae, and Schisandraceae) [9]–[14]. Previous studies on the families Araceae and Nelumbonaceae have focused on patterns of thermogenesis in terms of pollination biology, fertilisation and the biochemical regulation of heat production [15],[16], while only a few reports have linked thermogenic activity with the release of odours [2],[7],[17].

*Magnolia* species are early diverging angiosperms having thermogenic flowers with floral cycles of 2–4 days [18]–[20]. In these species, thermogenesis during anthesis commonly consists of two episodes, which are related to the male and female phases, respectively, in *Magnolia* species [3],[17],[21]. Floral fragrances are emitted during the anthesis of *Magnolia* [22],[23], and the scents are generally dominated by one chemical class of compounds, e.g. monoterpenes, benzenoids or hydrocarbons [23],[24]. In addition, pollination biology has been extensively studied, with beetles regarded as major pollinators [17],[22],[25]. Thermogenesis, floral scent emission and insect visitation occur simultaneously during anthesis, but our current knowledge of these phenomena in *Magnolia* is primarily derived from independent studies of thermogenesis patterns, floral scent and the species of visiting insects [22],[23],[26]. Very few combined studies have been performed to explore the relationship of thermogenesis with insect–plant interactions in Magnoliaceae or how thermogenesis is related to floral odour [3],[10],[17].

In our study, we focused on the association between thermogenesis and floral odour attractants in *Magnolia sprengeri*. Based on a description of the floral cycle, temperature measurements of floral thermogenicity and analyses of floral odour compounds, we investigated the visiting insects during different flowering stages and the relationships among floral thermogenesis, floral scent and pollinator visitation. The mutual relationships among thermogenic activity, variation in floral odour compounds and visiting frequency of pollinators during flowering uncovered in this study will increase our understanding of how Magnoliaceae ensures reproductive success at low temperatures in early spring.

## Materials and Methods

### Ethics statement

We obtained permission to perform this investigation from the Dalaoling National Nature Reserve Bureau, Hubei Province, China. No specific permission was required for this study since it did not involve any endangered or protected species in the sampled area.

### Study site and plant material

*Magnolia sprengeri* (Magnoliaceae) is an indigenous species distributed mainly in Shaanxi Province, west Hubei Province and the north-east part of Guizhou Province in China [27]–[29]. This study was conducted at Dalaoling Natural Reserve (30°50′–30°92′N, 110°52′–110°85′E) in Hubei Province, China. There are four distinct seasons in the study area, though spring and autumn are comparatively longer. The climate is moderate and humid, with 900–1500 mm of annual precipitation. In this population, *M. sprengeri* trees grow to 20–30 m in height and 20–30 cm in diameter at breast height. The trees typically flower from mid-March to the end of April, with peak blooming in mid-April.

### Determination of the floral cycle

This study was conducted from March 18 to April 26 of 2012. Movements of the petals, stigmas and anthers of individual flowers were recorded every day at anthesis for 30 flowers. The floral cycle was determined according to these floral characteristics.

### Insect visitation to flowers

Visiting insects were recorded during anthesis for 46 flowers on two trees (23 flowers per tree). This experiment was initiated on April 10, 2012. During the daytime, insects were collected in polyethylene bags (Reynolds, Lake Forest, IL, USA) from 10:00 to 18:30 at intervals of 1.5–2.0 h. At night, before the flowers closed to form a temporary chamber, we smeared Vaseline (Fuda Company, China), which is colourless, odourless and viscous, evenly to the basement of the inner petals to trap visiting insects. The trapped insects were collected the following morning. The insects collected were totalled by floral stages. As individual flowers had very small variation (<1.5 h) in the duration of anthesis and floral stages (Table 1), we divided the floral stages of the 46 flowers as follows: pre-pistillate stage, 10:00 day 1 (April 10) to 10:00 day 2; pistillate stage, 10:00 day 2 to 18:30 day 2; pre-staminate stage, 18:30 day 2 to 10:00 day 3; staminate stage, 10:00 day 3 to 18:00 day 3; and post-staminate stage, 18:30 day 3 to 10:00 day 4. All collected insects were sent for identification to the Beijing Natural History Museum or the Institute of Zoology of the Chinese Academy of Sciences.

**Table 1:** Description of the flowering stages of individual flowers of *Magnolia sprengeri*.

	Pre-pistillate stage	Pistillate stage	Pre-staminate stage	Staminate stage	Post-staminate stage
Petals	Folded tightly	Loose to open	Closed at night	Re-opened in the morning	Became brown
Stamens	Appressed tightly	Immature with anthers non-dehiscent	Loosened, but anthers do not dehisce	Radiated, all anthers dehisced	Became brown and abscising
Pistil	Opened slightly	Stigmas became receptive, arranged in order	Stigmas became appressed	Stigmas became appressed and unreceptive	Stigma withered
Starting time	day 1	10:00–11:00 on day 2	18:30–20:00 on day 2	10:00–11:00 on day 3	21:30–22:00 on day 3
Ending time	10:00–11:00 on day 2	18:30–20:00 on day 2	10:00–11:00 on day 3	21:00–21:30 on day 3	7:00–9:00 on day 4
Duration	22.5±1.2 h	8.5±0.9 h	15.2±1.2 h	11.2±0.8 h	10.2±1.0 h

As sap beetles and bees visited the flowers at both the pistillate and staminate stages (see results), suggesting that they were potential pollinators, we made additional observations of the visiting frequencies of sap beetles and bees to flowers at the pistillate and staminate stages. On April 15, 2012, 3 flowers at the pistillate stage and 3 flowers at the staminate stage were arbitrarily chosen in an individual tree. During the experiment, we made 0.5-h-long observations every 1.5 h. We recorded the number of sap beetles and bees landing on the chosen flowers.

### Floral thermogenesis

The floral temperature of 20 individual anthetic flowers on one tree was measured at 0.5-h intervals. A portable infrared thermal imaging radiometer [Ti55FT and FlexCam (≤0.5°C under 30°C); Fluke, Everett, WA, USA] was used to record the locations of flowers with the highest temperatures upon opening. Then, floral and ambient temperatures were recorded using thermocouples (0.3 mm in diameter) connected to a portable, battery-powered, digital thermometer (OS 685 L; Omega, Stamford, CT, USA) accurate to ±0.5°C. The thermocouples were inserted into the tissue at the basal part of the inner petals and gynoecium, which showed the highest temperature according to infrared thermal imaging radiometry (see the result). Each measurement was repeated three times, and the mean value was recorded. The air-temperature probe was hung freely at the same height. The flowers were shaded to avoid any influence from sunflecks and wind.

### Floral scents

Odour production was studied during the flowering period under natural conditions. Seven flowers were arbitrarily selected in the middle canopy of one tree, and floral scents were collected and analysed for each of the seven flowers during the five floral stages (10:00 for the pre-pistillate stage, 15:00 and 18:00 for the pistillate stage, 10:00 for the pre-staminate stage, 15:00 and 18:00 for the staminate stage and 08:00 for the post-staminate stage).

Floral odours were collected using the dynamic headspace technique [2]. Each flower was enclosed in a polyethylene bag (Reynolds), and the open end of the bag was bound around the stalk. The scent was collected in a glass tube containing 60 mg of Tenax-GR (mesh 80/100; CAMSCO, Houston, TX, USA) connected to the bag with an odourless silicone tube. Air was pumped at 200 ml/min. Circumambient air without flowers was collected as a control in the same manner. The trapped floral scents were stored in a freezer at −20°C before analysis. Gas chromatography–mass spectrometry (GC-MS) was performed using a TurboMatrix 650 ATD thermal desorber, and a Clarus 600 gas chromatograph (Perkin-Elmer, Waltham, MA, USA) coupled to a Clarus 600 mass spectrometer (Perkin-Elmer). The gas chromatographic column used was the DB-5MS (30 m×0.25 mm×0.25 µm; Clarus 600, Perkin-Elmer). The temperature was kept at 50°C for 8 min, programmed to 5°C/min to 150°C, and finally raised to 250°C at 10°C/min where it was kept for 8 min. The split value was 20∶1. Data analysis was performed using NIST 08 with TurboMass ver. 5.4.2.

### Data analysis

Floral scents were compared among stages by nonparametric multivariate analyses of variance (npMANOVA) [30] with the function adonis() from the vegan package in R [31] using the factors “flower”, “stage” and their interactions. If the npMANOVA revealed a significant effect of the factor “stage” on floral scents, *post hoc* tests were performed. *Post hoc* tests consisted of npMANOVAs with a Bonferroni correction. Such npMANOVA and *post hoc* tests were performed on the relative % of the three dominant compounds, then on the relative percentages of those compounds representing more than 1% of total blend.

## Results

### Determination of the floral cycle

**Figure 1:**
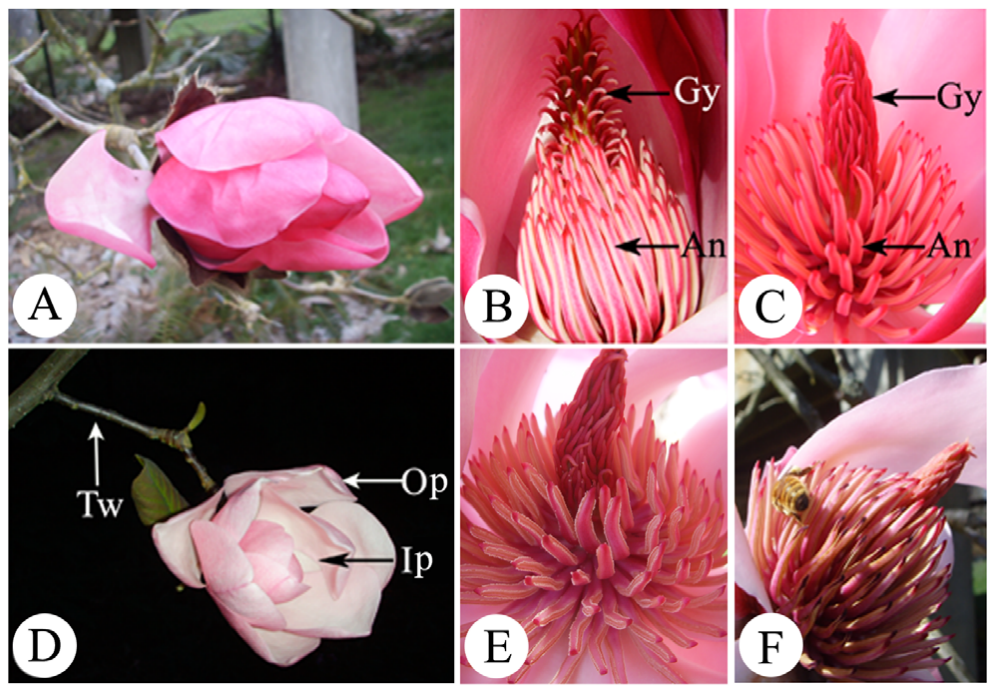
Morphology of an individual flower of *Magnolia sprengeri* at the pre-pistillate (A), pistillate (B), pre-staminate (C, D), staminate (E) and post-staminate (F) stages. (A) Pre-pistillate stage: petals folded tightly with pistils opened. (B) Pistillate stage: functionally female flowers with receptive stigmas and immature stamens with no dehiscent anthers. (C) and (D) Pre-staminate stage: a floral chamber with appressed and unreceptive stigmas and non-dehiscent anthers. (E) Staminate stage: functionally male flowers with gynoecium starting to fade; stamens detached from the axis and the anthers dehisced. (F) Post-staminate stage: withered flowers with the petals and stamens becoming brown and abscised; the stigma withered. Abbreviations: An, androecium; Gy, gynoecium; Ip, inner petals; Op, outer petals; Tw, twig.

Flowers of *M. sprengeri* appeared before the leaves, and were erect, cup-shaped and 15 cm wide with 12–14 petals that were white to rosy red in colour. The flowers were protogynous and the anthesis of an individual flower lasted for almost 4 days. According to the movements of the petals, stigmas and anthers of the flowers, the floral cycle could be divided into five distinct stages: pre-pistillate, pistillate, pre-staminate, staminate and post-staminate (Table 1). The pre-pistillate stage commenced 1 day before flower opening and ended in the following morning at 10:00–11:00. During this stage, the outer petals opened first, followed by the internal petals (Fig. 1A). Upon opening, the outer petals were initially held erect along with the inner petals, and then opened fully at 11:00 when the pistillate stage commenced. At the pistillate stage, the flowers were functionally female: the pistil was open and the receptive stigmas produced nectar-like exudates (Fig. 1B). The pistillate stage lasted until 18:30 in the afternoon. The flowers then closed to form a floral chamber (Fig. 1D), entering the pre-staminate stage. This stage was characterised by open stamens with non-dehisced anthers and a closed stigma (Fig. 1C) and lasted until 10:00–11:00 of the following morning. Then, the flower reopened, the surfaces of the stigmas shrank and the anthers began to dehisce, which indicated the start of the staminate stage (Fig. 1E). After about 11.5 h of pollen shedding, the stigmas began to turn brown and wither (Fig. 1F), which was indicative of the onset of the post-staminate stage.

### Floral pollinators

**Figure 2:**
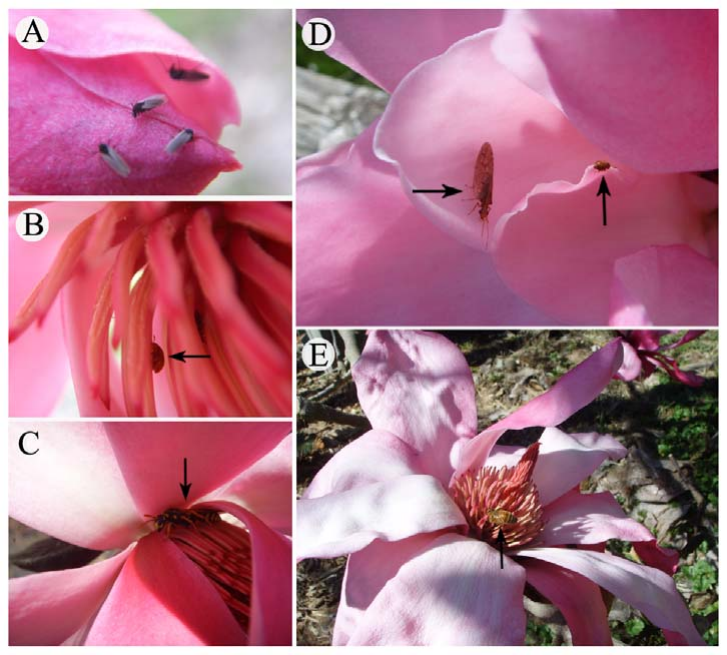
Flower-visiting insects at different stages of anthesis. (A) Aggregation of *Bibio* sp. on the surface of a swollen bud during the pre-pistillate stage. (B) *Epuraea* sp. foraging for pollen when the anthers were dehisced. (C) *Polistes chinensis* in a floral chamber at the staminate stage. (D) Brown lacewing (Hemerobiidae, Neuroptera) (left) and *Epuraea* sp. (right) crawling on the inner petals during the staminate stage. (E) *Apis cerana* foraging for pollen at the post-staminate stage.

The floral visitors to *M. sprengeri* included bibionid flies (*Bibio* sp., Bibionidae, Diptera), sap beetles (*Epuraea* sp., Nitidulidae, Coleoptera), bees (*Apis cerana*, Apidae, Hymenoptera), paper wasps (*Polistes chinensis*, Vespidae, Hymenoptera) and brown lacewings (Hemerobiidae, Neuroptera) (Table 2 and Fig. 2). Different insects visited the flowers at different floral stages (Table 2). The visiting frequencies of some insects varied during the anthesis. At the pre-pistillate stage, many bibionid flies (*Bibio* sp.) landed on the bud surfaces (Fig. 2A), particularly on partly exposed petals. However, they were absent during the pistillate stage; 187 sap beetles (*Epuraea* sp.) were observed both in the pistillate and staminate stages (Fig. 2B) and 89 of them landed on the pistillate-stage flowers and crawled on the stamens and gynoecia without foraging pollen (no pollen was produced during the pistillate stage). During the staminate stage, we recorded 72 sap beetles; some of them stayed inside the flowers for longer than 30 min and then flew into the loose stamens. We observed pollen on their bodies in both the pistillate and staminate stage flowers (Fig. 2B). Therefore, they were considered to be the most effective pollinators of *M. sprengeri*.

**Table 2:** Number of floral visitors to 46 *Magnolia sprengeri* flowers observed during the five floral stages.

	Pre-pistillate	Pistillate	Pre-staminate	Staminate	Post-staminate
Bibionidae					
*Bibio* sp.	62	0	0	3	5
Nitidulidae					
*Epuraea* sp.	0	89	23	72	3
Vespidae					
*Polistes chinensis*	0	0	0	5	2
Apidae					
*Apis cerana*	0	8	0	21	2
Hemerobiidae	0	0	2	2	2

**Figure 3:**
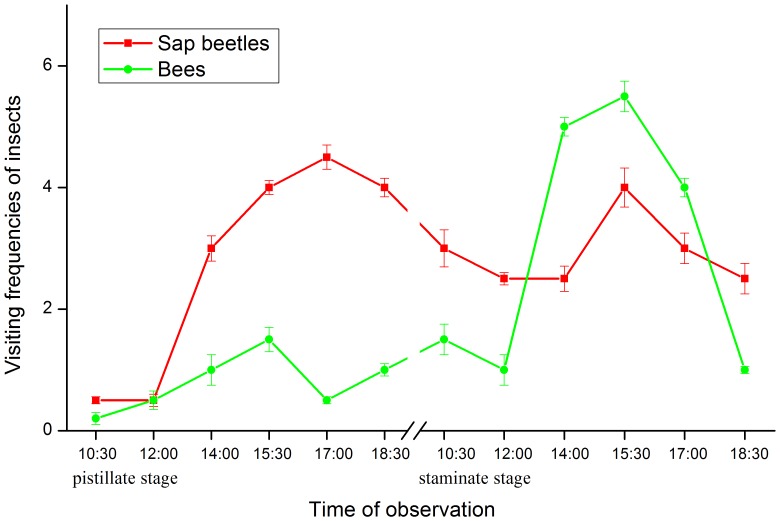
Visiting frequencies of sap beetles and bees on *Magnolia sprengeri* flowers at pistillate and staminate stages. The observation was conducted on 6 flowers. Sap beetles and bees landing on the observed flowers for half an hour were recorded at an interval of 1.5

In addition, bees (*A. cerana*) also visited flowers at both the pistillate and staminate stages; however, when compared with sap beetles, far fewer bees were observed at the pistillate stage (Fig. 3). Thus, they were considered to be occasional pollinators. The majority of flies (*Bibio* sp.) were observed on the surface of swollen buds during the pre-pistillate stage; a small number of paper wasps (*P. chinensis*) and brown lacewings (Hemerobiidae) also foraged at the staminate and post-staminate stages (Fig. 2C and D), but none of them visited pistillate-stage flowers. Thus, these insects were not considered effective pollinators.

### Floral thermogenesis

**Figure 4:**
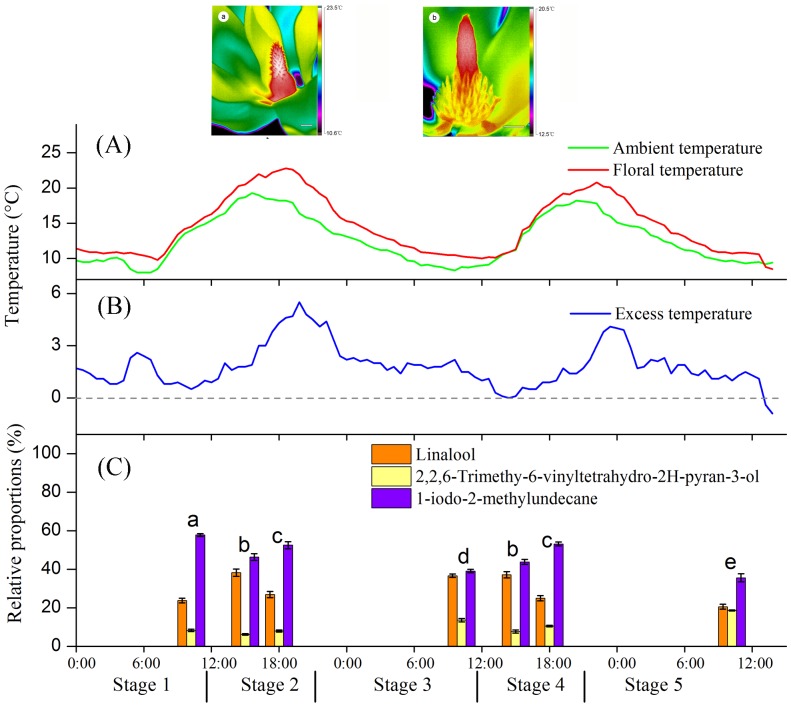
Changes in temperatures and floral scents of *Magnolia sprengeri* during anthesis. (A) Records of *Magnolia sprengeri* flower temperature (red line) and ambient air temperature (green line) during anthesis. (B) Difference between flowers and air temperature. (C) Relative proportions of dominant compounds (contributing to >5% of the blend) of *M. sprengeri* floral scents during the five flowering stages*. In (A), pictures a and b are infrared images of a *M. sprengeri* flower during the pistillate (a) and the staminate stages (b). Bar scales represent 1.5 cm in image a and 2 cm in image b. Colours indicate the temperature distribution. The red colour in the centre of the flower corresponds to the highest temperature. In (B), the same lowercase letters indicate no significant difference (p>0.05) in odour compounds between the floral stages according to the npMANOVA post hoc tests. Error bars indicate standard errors. Stage 1, pre-pistillate stage; Stage 2, pistillate stage; Stage 3, pre-staminate stage; Stage 4, staminate stage; Stage 5, post-staminate stage. * Relative proportions of chemical compounds accounting to >1% of the total floral scents differed significant (p<0.05) among all 7 samples.

Thermogenesis in *M. sprengeri* consisted of two distinct peaks (Fig. 4). The first peak occurred between 12:00 and 18:30 on the second day of anthesis, corresponding with the pistillate stage when stigmas were receptive (Fig. 4). During this peak, the floral temperature was 1.0–5.5°C above the ambient air temperature (Fig. 4). The second peak occurred between 14:00 and 18:30 in the third day of anthesis. This peak was lower (0.9–4.1°C) and synchronised with pollen liberation and dispersion during the staminate stage.

### Floral scent variation during different stages of flowering

*Magnolia sprengeri* flowers produced a strong fragrance. Floral scent analyses were conducted during the four developmental stages (Table 3). In total, 18 compounds were identified in the floral scents. Three compounds, linalool, 1-iodo-2-methylundecane and 2,2,6-trimethyl-6-vinyltetrahydro-2H-pyran-3-ol (epoxylinalool), accounted for more than 5% of the total scent of each sample (Table 3). These compounds were considered dominant compounds.

**Table 3:** Retention time and relative proportions (%) of chemical compounds identified in scents collected on 7 *M. sprengeri* flowers at different flowering stages (values in this table are means±s.e.).

	Retention time (min)	Pre-pistillate stage	Pistillate stage		Pre-staminate stage	Staminate stage		Post-staminate stage
			A	B		A	B	
Heptanal	5.67	0.22±0.08	-	-	-	0.53±0.02	-	1±0.08
Acetophenone	7.05	0.11±0.05	0.18±0.07	0.22±0.01	-	-	-	-
Methyl heptenone	7.66	-	-	-	0.20±0.01	-	-	-
Myrcene	7.74	0.12±0.03	0.28±0.08	0.74±0.03	0.18±0.01	0.31±0.02	0.27±0.06	0.33±0.05
Caprylic aldehyde	8.09	-	-	-	-	-	0.11±0.03	-
Cyclohexene	8.67	0.33±0.08	0.22±0.09	0.32±0.03	0.47±0.01	0.74±0.01	-	0.57±0.02
Pelargonic aldehyde	10.62	1.25±0.15	-	0.64±0.02	0.95±0.02	0.22±0.01	0.92±0.19	-
Linalool	10.64	23.73±1.2	38.25±1.87	26.89±0.60	36.62±0.60	37.13±0.57	24.95±1.32	20.53±1.27
Phenylethanol	10.95	1.15±0.25	1.72±0.19	1.40±0.02	0.76±0.02	1.06±0.01	-	0.48±0.12
Tridecylic aldehyde	12.19	-	-	-	-	-	0.21±0.06	-
epoxylinalool	12.29	8.25±0.60	6.22±0.39	7.91±0.49	13.57±0.53	7.67±0.31	10.51±0.42	18.67±0.28
(L)-Alpha-terpineol	12.80	1.97±0.18	1.21±0.15	1.30±0.01	1.12±0.02	0.70±0.01	1±0.17	1.15±0.08
Decyl aldehyde	13.04		-	-	-	-	0.41±0.08	-
Indole	14.98	1.07±0.21	0.71±0.15	2.24±0.02	1.87±0.03	1.21±0.02	1.84±0.18	5.18±0.09
Undecyl aldehyde	15.35	-	-	-	-	0.27±0.01	-	-
2-Phenylethyl 2-methylbutyrate	19.10	1.93±0.25	1.83±0.25	3.25±0.02	2.35±0.03	2.05±0.02	3.29±0.19	3.59±0.18
Alpha-farnesene	19.23	2.11±0.19	3.1±0.21	2.60±0.13	2.86±0.20	4.32±0.12	3.35±0.21	12.95±0.35
1-Iodo-2-methylundecane	19.55	57.77±0.72	46.27±1.75	52.48±0.87	39.05±0.81	43.79±0.83	53.13±0.97	35.56±2.10

Note: The floral odours were sampled at 10:00 for the pre-pistillate stage, 15:00 (A) and 18:00 (B) for the pistillate stage, 10:00 for pre-staminate stage, 15:00 (A) and 18:00 (B) for the staminate stage, and 8:00 for the post-staminate stage.

As expected, floral stage was an important factor influencing the relative amount of compounds in floral scents (p = 0.001; Table 4). Table 4 clearly shows that no significant difference in the odour compounds was detected among the seven flowers sampled (p = 0.558). In addition, the interaction between “floral stage” and “flower” showed no significant cross effect on the compounds within floral scents (p = 0.922; Table 4), meaning that the variation in odour between the stages was the same for each flower. According to the *post hoc* tests, the scent composition of different floral stages was significantly different (Fig. 4C). With regard to the dominant compounds, the scents sampled at 15:00 during the pistillate stage were not significantly different to those sampled at 15:00 during the staminate stage. Such similarity was also found in floral scents sampled at 18:00 during the pistillate and staminate stages (Fig. 4C).

**Table 4:** Summary of the npMANOVA for the odour compounds of seven flowers sampled at the five floral stages. d.f.  =  degrees of freedom.

	d.f.	F	R^2^	p
A				
Stage*	6	14.88	0.246	0.001
Flower	6	0.59	0.010	0.558
Stage × Flower	36	0.09	0.002	0.922
B				
Stage*	6	17.06	0.272	0.001
Flower	6	0.57	0.009	0.579
Stage × Flower	36	0.09	0.001	0.952

*Although only five floral stages occurred, floral scents were sampled twice at both the pistillate and staminate stages and once at the pre-pistillate, pre-staminate and post-staminate stages. Thus, the factor “stage” had a d.f. of 6 (i.e. 7–1). A. Analysis performed on the three dominant compounds from the blends. B. Analysis performed on all compounds accounting for more than >1% of the blend.

## Discussion

Similar to other early diverging angiosperms [12],[19],[32], the flowers of *M. sprengeri* are bisexual, protogynous, fragrant and pollinated by insects (especially beetles). When the flowers opened for the first time during the daytime, they were functionally female with a receptive stigma and non-dehiscent stamen. The flowers then closed overnight, forming floral chambers, until their reopening the following morning. In some thermogenic plants, e.g. *Philodendron solimoesense* and *Symplocarpus foetidus*, heated floral chambers provide shelter for beetle pollinators where they can mate, feed and pollinate the flowers at night during cold periods, and benefit from the energy provided by the flower's heat in the cool mornings [6],[33],[34]. In our study, some sap beetles remained inside the floral chambers at night during the pre-staminate stage. We thus suggest that *M. sprengeri* flowers may act as shelter for sap beetles during the cold night.

Heat production in gynoecia of *M. sprengeri* flowers occurred during anthesis and consisted of two distinct peaks. Distinct heating patterns have been reported in different thermogenic species [35],[36]. In some thermogenic plants, floral temperature is maintained at a constant range, independent of ambient temperature, for a long period throughout anthesis [8],[35],[36]. Floral temperature stability of these plants is achieved by means of physiological thermoregulation [35],[36]. In this study, floral temperature of *M. sprengeri* changed constantly throughout anthesis and major thermogenic episodes were short, which indicated that *M. sprengeri* was not thermoregulated. Our result is in agreement with a previous study on floral thermogenesis of *M. ovata* in Brazil [3].

Thermogenesis is generally linked to volatilisation of floral scent [37]. Like many other *Magnolia* species [23], floral scent was emitted during the thermogenesis of *M. sprengeri*. Floral scents have been well documented to play an important role in plant–pollinator interactions through various effects on visiting insects, e.g. as a feeding cue and nectar guide [38]. Beetles are important pollinators of many early diverging angiosperms, including *Magnolia* [32]. In this study, we found that many sap beetles (*Epuraea* sp.) visited *M. sprengeri* flowers at both the pistillate and staminate stages and were identified as effective pollinators. When the sap beetles were in the flowers, they were frequently observed foraging pollen grains during the staminate stages. We thus concluded that the floral odours of *M. sprengeri* may act as a signal of food resources for sap beetles.

Floral scents are complexes of chemical compounds [39], and different odours are selected by different insects [2],[40]. Intriguingly, we found a similarity in dominant compounds of floral scents at pistillate and staminate stages in *M. sprengeri*. In a previous study, Gottsberger *et al.* also reported a smell similarity between pistillate and staminate stage flowers of *M. ovata* [17]. In addition, it has been hypothesized that staminate stage flowers mimic pistillate stage flowers in *M. hypoleuca*, as the former offers no pollen reward but has strong fragrance [19]. Our study validates odour mimicry between the female and the male stage flowers in a *Magnolia* species by experimental data. As no food reward for pollinators exists at the female stage, this mimicry might be an adaptive strategy for *Magnolia* species to attract pollinators at both stages, ensuring successful pollination.

Patterns of heating may also be a crucial aspect of the adaptive strategies of *M. sprengeri*. Because thermogenesis promotes the volatilization of floral scents, which are cues for insects signifying the presence of food resources, the production of heat by thermogenic plants when pollinators are active might be a more “efficient” strategy. We found that this species flowers in early spring and their flowers produce heat in daytime, which is well synchronised with the activity of its pollinator (sap beetles, *Epuraea*, Nitidulidae) [41],[42]. In some other *Magnolia* species (e.g. *M. tamaulipana* [22] and *M. ovata* [3],[17]), thermogenesis peaks occur at night. As reported previously, *M. tamaulipana* and *M. ovata* generally grow in wet and warm habitats occupied by *Cyclocephala* (Dynastinae, Scarabaeidae), which are active at night [17],[22]. Considering these results, one can reasonably conclude that the divergence in thermogenesis patterns among various *Magnolia* species is linked to the activity of their pollinators, but further evidence is needed to support this idea.
